# Factors associated with body size perception among adolescent goal-oriented sports participants and non-participants: a cross-sectional Finnish study

**DOI:** 10.1186/s12889-022-14511-z

**Published:** 2022-11-25

**Authors:** Leevi Mäkituomas, Laura Heikkilä, Marjukka Nurkkala, Raija Korpelainen, Lauri Alanko, Olli J. Heinonen, Sami Kokko, Urho Kujala, Jari Parkkari, Kai Savonen, Maarit Valtonen, Tommi Vasankari, Jari Villberg, Marja Vanhala

**Affiliations:** 1 Department of Sports and Exercise Medicine, Oulu Deaconess Institute Foundation sr., P.O. Box 365, FI-90101 Oulu, Finland; 2 Center for Life Course Health Research, Faculty of Medicine, University of Oulu, P.O. Box 5000, FI-90014 Oulu, Finland; 3 Medical Research Center, University of Oulu and Oulu University Hospital, P.O. Box 8000, FI-90014 Oulu, Finland; 4 Sports Medicine Clinic, Foundation for Sports and Exercise Clinic, Mäkelänkatu 47, FI-00550 Helsinki, Finland; 5 Sports and Exercise Medicine Clinic, Central Finland Hospital Nova, Hoitajantie 3, FI-40620 Jyväskylä, Finland; 6 Paavo Nurmi Centre & Unit for Physical Activity and Health, University of Turku, Kiinamyllynkatu 10, FI-20520 Turku, Finland; 7 Faculty of Sport and Health Sciences, University of Jyväskylä, P.O. Box 35, FI-40014 Jyväskylä, Finland; 8 Tampere Research Center of Sports Medicine, Kaupinpuistonkatu 1, FI-33500 Tampere, Finland; 9 Kuopio Research Institute of Exercise Medicine, Haapaniementie 16, FI-70100 Kuopio, Finland; 10 Department of Clinical Physiology and Nuclear Medicine, Science Service Center, Kuopio University Hospital, P.O. Box 100, FI-70029 KYS, Kuopio, Finland; 11 Research Institute for Olympic Sports, Rautpohjankatu 6, FI-40700 Jyväskylä, Finland; 12 UKK Institute of Health Promotion Research, Kaupinpuistonkatu 1, FI-33500 Tampere, Finland; 13 Faculty of Medicine and Health Technology, Tampere University, Kalevantie 4, FI-33014 Tampere, Finland

**Keywords:** Body satisfaction, Organized youth sports, Competitive sports, Athlete, Cross-sectional

## Abstract

**Background:**

Regardless of competitive athletes’ body image pressures, only few studies have focused on adolescent sport participants’ body image and the findings are inconclusive. Furthermore, the role of competitive goals in sports on adolescents’ body size perception has not been studied. We examined the factors associated with adolescents’ competitive goals in sports and body size perception, and the associations between adolescents’ competitive goals in sports and body size perception.

**Methods:**

The cross-sectional study consisted of 475 goal-oriented sports club participants and 936 reference youths (aged 14–16 years). The study questionnaire included multiple choice items on health behaviours, motives to exercise, competitive goals in sports and body size perception. The multinomial logistic regression analysis was used to investigate the associations.

**Results:**

Adolescents with competitive goals in sports perceived their body size as about the right size more frequently than reference youths (68% vs 47%, *p* < 0.001 in girls; 74% vs 61%, *p* < 0.001 in boys). More than one-fourth of girls with competitive goals in sports perceived themselves as overweight, although only 7% of them were overweight. Adolescents with appearance/weight motives to exercise and poor perceived physical fitness had higher odds of perceived fatness. Additionally, BMI was positively associated with perceived fatness and negatively with perceived thinness. Having competitive goals in sports was not independently associated with perceived fatness or perceived thinness.

**Conclusions:**

Adolescents’ BMI, appearance/weight motives to exercise, and perceived physical fitness were more strongly associated with body size perception than their competitive goals in sports. However, perceived fatness among girls with competitive goals in sports should be considered in organized sports.

**Supplementary Information:**

The online version contains supplementary material available at 10.1186/s12889-022-14511-z.

## Background

Body image has been defined as an individual’s thoughts, perceptions, feelings, and beliefs about his or her body [1]. Adolescence is a critical period for body image development because of the various physical, psychological, and sociocultural changes occurring during that period [2]. The prevalence of adolescents’ body weight dissatisfaction has been shown to range between 18 and 57% [3]. Body dissatisfaction is mainly thought to originate from pressures to comply with sociocultural ideals of appearance that are virtually unattainable for most as well as internalization of these ideals [2, 4]. Increased body dissatisfaction weakens self-esteem and has been associated with the development of eating disorders and extreme weight-loss behaviours [5, 6].

According to a recent meta-analysis among females, athletes have lower levels of body dissatisfaction than non-athletes [7]. A previous meta-analysis proposed athletes’ lower body image disturbance be due to athletes resembling the cultural aesthetic ideal more than non-athletes or due to the positive effect of physical activity on self-esteem [8]. Higher levels of functionality appreciation among athletes than non-athletes can also explain their more positive body image [9]. Despite of lower body dissatisfaction, the prevalence of eating disorders has been reported to be higher among elite athletes than adolescents and adults in general [10]. Differences in disordered eating behaviour have been suggested to relate to, for example, athletes’ personality, such as high achievement orientation and perfectionism, and coaching behaviours [11, 12].

Findings on athletes’ body image have varied between competition levels and sport types [13–15]. Competitive athletes can experience the same pressures to reach body ideals than non-athletes, but also have coach and uniform pressures and teammate influences on body image [15, 16]. Highly competitive female athletes may also be more likely to struggle with achieving both athletic and thin body ideals [13]. However, only few studies have focused on adolescent sport participants’ body image and the findings are inconclusive [17–19]. One recent study found that adolescents’ sports participation was related to higher body image satisfaction, but differences were not found between competitive athletes, leisure athletes, and non-athletes in body size perception (BSP) [18].

Sport specialization and assessment of the desired level of competition are usually topical in adolescence. Although findings on athletes’ body image have varied by competition level [13, 15], the role of competitive goals in adolescent sports club participants’ BSP has not previously been examined. Furthermore, despite adolescence is a pivotal stage in body image development, most of the studies concerning athletes’ body image and competition level have been conducted in older adolescents or adults [13, 15].

The present study aimed to examine the factors associated with adolescents’ competitive goals in sports and BSP as well as the associations between adolescents’ competitive goals in sports and BSP among a representative sample of Finnish sports club participants and non-participants. It was hypothesized that adolescent sports club participants with competitive goals in sports are more likely to perceive themselves as about the right size than non-goal-oriented sports club participants and non-participants.

## Methods

### Study setting and design

This cross-sectional study was part of the ongoing longitudinal Finnish Health Promoting Sports Club study [20]. The baseline data used in this study were collected in 2013–2014. The study was conducted in accordance with the Declaration of Helsinki. Ethical approval was received from the Ethics Committee of Health Care District of Central Finland (record number 23 U/2012).

For the questionnaire data, written informed consent was received from the participating youth and their guardians were informed about their child’s participation in the study. According to the approval of the Ethics Committee of Health Care District of Central Finland, written or verbal consent was not required from the guardians for the questionnaire data. For the accelerometer-measured physical activity data, written informed consent was obtained from a guardian and the adolescent him/herself.

The study protocol has been described in detail earlier [20]. In short, sports club participants were recruited via sports clubs in two stages. First, the clubs were stratified depending on 1) winter and summer sports (according to the main competition season) and team and individual sports, and 2) club’s magnitude, geographical location, area type, and certification by the Young Finland Association. Ten most popular sports disciplines in Finland were included in the study: basketball, cross-country skiing, floorball, gymnastics, ice hockey, orienteering, skating, soccer, swimming, and track and field. Twenty-four sports clubs from each of the sports disciplines were targeted and 156 clubs took part.

In the second stage, sports club participants aged 14–16 years were invited to the study. Individual sports participants were randomly selected from name lists provided by clubs. Of the team sports clubs, one team was randomly chosen, and the researchers randomly selected the individuals from team participant name lists. In total, 1889 sports club participants were invited and 759 (40%) of them completed the study questionnaire.

Similarly, the age-matched sample of non-participants was collected via schools in two stages. First, ten secondary schools from each of six different areas in Finland were targeted, and the schools were stratified depending on school’s magnitude and area type. Second, one randomly selected class of ninth graders from each school was asked to respond to the questionnaire. Overall, 2074 pupils were invited from one hundred schools and 1650 (80%) of them responded to the study questionnaire.

Adolescents with missing information about age, who were not 14–16 years old, who had inadequate information about weight or height, or who had contradicting answers regarding sports club participation, duplicate answers, improper answers, or who forbade the use of data for research were removed from the data (166 sports club participants and 285 pupils). The school-based sample included both sports club participants and non-participants. The questionnaire of the school-based sample did not include the competitive goal question. Hence, pupils participating in sports clubs (*n* = 547) were not included.

In total, 593 adolescents via sports clubs and 818 adolescents via schools participated in the study. Sports club participants who did not have competitive goals in sports (*n* = 118) were included in the reference group in the analyses. Therefore, the competitive goals in sports (CGS) group comprised 475 participants and the reference group consisted of 936 participants.

### Study questionnaire

The data were collected using two Internet-based questionnaires. Sports club participants replied to the questionnaire on their spare time and pupils in class during a school day. Whether participants had competitive goals in sports were assessed by the question: “What is your competitive goal as an athlete?” Adolescents who aimed to succeed in junior competitions (at the regional, national, or international level) or adult competitions (at the international or national level) were considered as the CGS group. Those who replied that “I have no competitive goals, I exercise to develop in sports” or “I have no competitive goals, I exercise in a recreational sense” formed the reference group.

Body size perception was assessed with the question described in the Health Behaviour in School-aged Children study [21]: “Do you think your body is [much too thin / a bit too thin / about the right size / a bit too fat / much too fat]?” The test-retest stability of the question has been good among Finnish adolescents [22]. The initial variable with five categories was re-coded into a three-class variable, where the “a bit too thin” and “too thin” categories and the “a bit too fat” and “too fat” categories were combined. “Too thin” and “too fat” were considered as the examined responses and “about the right size” was a reference category.

The motives to exercise were investigated by a modified version of the Nigg’s questionnaire [23]: “Are the following issues important for your exercising [not important / somewhat important / very important]?” The answers were re-coded into binomial variables with answer options “very or somewhat important” and “not important”. The motives to exercise were categorized into four categories [24, 25]:

- *appearance/weight* (to look good, to control weight, to lose weight)
- *health/fitness* (to improve health, to improve physical fitness, to gain muscle)
- *social* (to win, to be good in sports, to make new friends, to meet my friends, to please my parents, to be “cool”)
- *enjoyment* (to have fun, to appreciate the sensations during exercise, exercise is exhilarating)

For further analysis, initial responses were scored as: not important = 0, somewhat important = 1, and very important = 2. The values were summed (range 0–12 for social motives and 0–6 for other motives) and a higher score indicated a higher motive.

Self-rated health and perceived physical fitness were separately assessed by the question: “Would you say your health/physical fitness is… [excellent / good / fair / poor]?” The responses were dichotomized as “good” (excellent/good) and “poor” (fair/poor). BMI was calculated from self-reported height and weight data. Age- and sex-specific cut-off points for adolescents’ BMI were based on the international growth reference data [26, 27]. Cut-off points for underweight individuals were set for BMI < 17 kg/m^2^, for normal weight at BMI = 17–25 kg/m^2^, and for overweight at BMI > 25 kg/m^2^.

### Accelerometer-measured physical activity

**Fig. 1:**
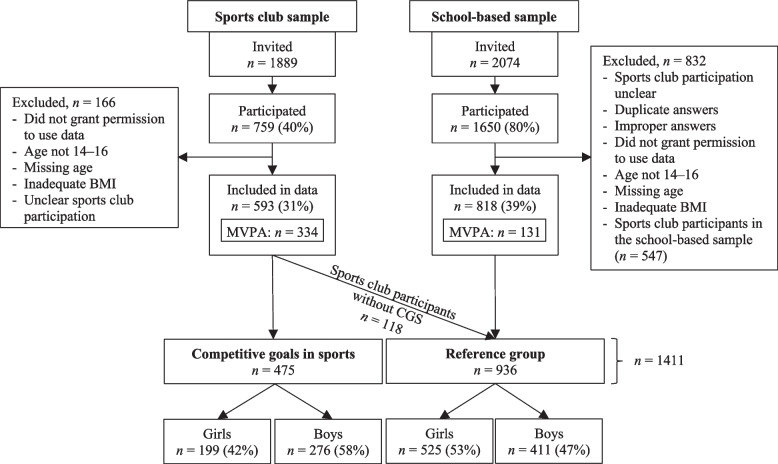
Sampling of study participants [20]. BMI = body mass index; CGS = competitive goals in sports; MVPA = moderate to vigorous physical activity

Moderate to vigorous physical activity (MVPA) was measured from a randomly selected sample of sports club participants (*n* = 334) and non-participants (*n* = 131) (Fig. 1). Adolescents were instructed to use a hip-worn, light tri-axial accelerometer (AM20 Activity Meter, Hookie Technologies Ltd., Helsinki, Finland) for seven consecutive days during waking hours with the exception of during shower or water activities. Swimmers (*n* = 31) were excluded as they were unable to use the accelerometer during water activities. The device has been shown to be a valid measurement tool for continuous monitoring of physical activity among both youths [28] and adults [29, 30].

The tri-axial data were collected with a sampling frequency of 100 Hz. The mean amplitude deviation (MAD) of the resultant was analysed in 6 s epoch length and converted to metabolic equivalents (MET). Cut-points were set as 3.0–6.0 MET for moderate activity and over 6.0 MET for vigorous activity. The mean daily moderate and vigorous activities were summed to obtain the amount of MVPA.

### Statistical analyses

All the statistical analyses were stratified by gender. The descriptive analyses were performed using cross-tabulation and χ^2^ test for categorical variables. For continuous normally distributed variables, the independent samples *t* test and one-way ANOVA were used. For non-normally distributed variables, Mann Whitney U test and Kruskal-Wallis test were employed. The multinomial logistic regression models were built to assess the associations between competitive goals in sports and BSP: “too fat” and “too thin” were used as the examined responses and “about the right size” was a reference category. The models were controlled for competitive goals in sports, BMI, perceived physical fitness, self-rated health, appearance/weight motives, enjoyment motives, health/fitness motives, social motives, and MVPA. Statistical analyses were performed using SPSS version 25 (Armonk, New York, IBM Corp.). The significance level was set at *p* < 0.05.

## Results

### Competitive goals in sports

We compared adolescent competitive goal-oriented sports club participants (*n* = 475) to the reference group including non-goal-oriented sports club participants (*n* = 118) and non-sports club participants (*n* = 818). Adolescents in the competitive goals in sports (CGS) group and in the reference group were of similar age, weight, and height, except CGS girls were taller than other girls (Table 1). CGS youths were more likely to perceive their health and physical fitness as good and had higher accelerometer-measured MVPA than reference youths.

**Table 1:** Characteristics of the study groups by competitive goals in sports

Characteristics	Girls (*n* = 724)				Boys (*n* = 687)			
	CGS (*n* = 199)		RG (*n* = 525)		CGS (*n* = 276)		RG (*n* = 411)	
Age (years), mean (SD)	14.9	(0.7)	14.9	(0.5)	15.0	(0.5)	15.0	(0.5)
Weight (kg), mean (SD)	58.1	(7.7)	57.5	(11.0)	64.4	(9.3)	65.2	(11.5)
Height (cm), mean (SD)	166.8	(6.4)	164.9	(6.2)***	175.2	(7.3)	174.6	(7.8)
BMI (kg/m2), mean (SD)	20.8	(2.1)	21.1	(3.5)	20.9	(2.2)	21.4	(3.2)*
Normal BMI^a^, n (%)	185	(93.0)	429	(81.7)	243	(88.0)	318	(77.4)
BMI < 17^a^, n (%)	1	(0.5)	7	(1.3)	0	(0.0)	1	(0.2)
BMI > 25^a^, n (%)	13	(6.5)	89	(17.0)***	33	(12.0)	92	(22.4)**
Self-rated good health, n (%)	191	(96.0)	407	(77.5)***	272	(98.6)	340	(82.7)***
Perceived good physical fitness, n (%)	181	(91.0)	272	(51.8)***	265	(96.0)	284	(69.1)***
MVPA (min/d)^b^, mean (SD)	83	(27)	60	(21)***	104	(33)	74	(32)***
*Body size perception*								
About the right size, n (%)	135	(67.8)	244	(46.5)***	204	(73.9)	250	(60.8)***
Too thin, n (%)	10	(5.0)	39	(7.4)	56	(20.3)	74	(18.0)
Too fat, n (%)	54	(27.1)	242	(46.1)***	16	(5.8)	87	(21.2)***
*Motives to exercise*								
Appearance/weight motives, median (IQR)	3.0	(3.0)	4.0	(3.0)***	2.0	(2.0)	3.0	(2.0)
Health/fitness motives, median (IQR)	5.0	(2.0)	5.0	(2.0)*	5.0	(2.0)	4.0	(3.0)***
Social motives, median (IQR)	7.0	(3.0)	5.0	(3.0)***	7.0	(3.0)	6.0	(3.0)***
Enjoyment motives, median (IQR)	6.0	(1.0)	4.0	(3.0)***	5.0	(2.0)	4.0	(2.0)***

*BMI* body mass index, *CGS* competitive goals in sports, *IQR* interquartile range, *MVPA* moderate to vigorous physical activity, *RG* reference group

^a^Classification based on the international BMI cut-off points [26, 27]

^b^Measured using a accelerometer from a randomly selected sample of adolescents (241 girls and 193 boys)

*P* values are presented for differences between CGS and reference groups: *p < 0.05, **p < 0.01, ***p < 0.001

CGS youths were more likely to perceive themselves as about the right size than reference youths, whereas the proportion of normal weight adolescents was similar between the groups. Adolescents in the CGS group were less likely to perceive themselves as too fat and less frequently overweight compared with the reference group. There was no difference between the CGS and reference groups in those perceiving themselves as too thin.

Among girls, the ratio of perceived fatness and being overweight was higher in the CGS group than it was in the reference group (4.2 vs 2.7, respectively). More than one-fourth of girls with CGS perceived themselves as too fat, although only 7% of them were actually overweight. In the reference group, about half of girls perceived fatness and 17% were overweight. Among boys, the ratio was lower in the CGS group compared with the reference group (0.5 vs 0.9, respectively). None of the boys with competitive goals in sports were underweight, but every fifth boy perceived himself as too thin. In terms of motives to exercise, CGS youths more frequently reported health/fitness, social, and enjoyment motives, and fewer CGS youths than reference youths exercised to lose weight in both genders (Supplementary Table 1).

### Body size perception

Girls perceived their body as about the right size less often than boys (52% vs 66%, *p* < 0.001) and were more likely to consider themselves too fat (41% vs 15%, *p* < 0.001; Table 2). Most girls who did not perceive themselves as about the right size considered themselves too fat (86%), whereas boys with body size dissatisfaction more equally perceived fatness or thinness (44 and 56%, respectively).

**Table 2:** Characteristics of the study participants by body size perception

Characteristics	Girls (n = 724)						Boys (n = 687)					
BSP, n (%)	Too thin^a^		About the right size		Too fat^b^		Too thin^a^		About the right size		Too fat^b^	
	49	(6.8)	379	(52.3)	296	(40.9)	130	(18.9)	454	(66.1)	103	(15.0)
BMI (kg/m2), mean (SD)	18.0	(2.2)***	20.0	(2.0)	22.8	(3.6)***	19.5	(2.0)***	20.9	(2.2)	24.4	(3.4)***
BMI < 17^c^, n (%)	6	(12.2)***	2	(0.5)	0	(0.0)	1	(0.8)	0	(0.0)	0	(0.0)
Normal BMI^c^, n (%)	42	(85.7)	359	(94.7)	213	(72.0)	122	(93.8)	397	(87.4)	42	(40.8)
BMI > 25^c^, n (%)	1	(2.0)	18	(4.7)	83	(28.0)***	7	(5.4)*	57	(12.6)	61	(59.2)***
Competitive goals in sports, n (%)	10	(20.4)*	135	(35.6)	54	(18.2)***	56	(43.1)	204	(44.9)	16	(15.5)***
Self-rated good health, n (%)	38	(77.6)*	350	(92.3)	210	(70.9)***	114	(87.7)*	423	(93.2)	75	(72.8)***
Perceived good physical fitness, n (%)	30	(61.2)*	288	(76.0)	135	(45.6)***	105	(80.8)	396	(87.2)	48	(46.6)***
MVPA (min/d)^d^, mean (SD)	81	(26)	75	(27)	65	(25)*	92	(38)	97	(34)	63	(28)**
*Motives to exercise*												
Appearance/weight motives, median (IQR)	2.0	(3.0)	3.0	(2.0)	5.0	(2.0)***	2.0	(2.0)*	2.0	(2.0)	3.0	(2.0)***
Health/fitness motives, median (IQR)	5.0	(3.0)	5.0	(1.0)	5.0	(2.0)	5.0	(3.0)	5.0	(2.0)	4.0	(3.0)
Social motives, median (IQR)	6.0	(3.0)	6.0	(3.0)	5.0	(3.0)	6.0	(4.0)	6.0	(3.0)	6.0	(4.0)**
Enjoyment motives, median (IQR)	4.0	(3.0)	5.0	(2.0)	4.0	(3.0)**	5.0	(3.0)	5.0	(2.0)	4.0	(2.0)**

*BMI* body mass index, *BSP* body size perception, *IQR* interquartile range, *MVPA* moderate to vigorous physical activity

^a^*P* values for difference between the groups “too thin” and “about right size”: *p < 0.05, ***p < 0.001

^b^*P* values for difference between the groups “too fat” and “about right size”: *p < 0.05, **p < 0.01, ***p < 0.001

^c^Classification based on the international BMI cut-off points [26, 27]

^d^Measured using a accelerometer from a randomly selected sample of adolescents (241 girls and 193 boys)

BMI increased according to body size perception categories. The adolescents who perceived their body too fat more often rated their health and physical fitness as being poor than did those who perceived their bodies to be about the right size. Perceived fatness was also negatively associated with MVPA. Perceived fatness was associated with greater emphasis on exercise for appearance/weight management and smaller importance placed on enjoyment motives. Adolescents who perceived themselves too thin less frequently self-rated their health as good and exercised to lose or control weight (Supplementary Table 2).

### Multivariable models

Perceived fatness was positively associated with BMI and appearance/weight motives to exercise, and negatively with perceived physical fitness among all participants (Table 3). In addition, girls who perceived themselves as too fat were less likely to have health/fitness motives to exercise. Perceived thinness was negatively associated with BMI. Having competitive goals in sports was not associated with perceived fatness (*p* = 0.53 in girls; *p* = 0.11 in boys) or perceived thinness (*p* = 0.77 in girls; *p* = 0.52 in boys).

**Table 3:** Multinomial logistic regression models for (a) girls and (b) boys on the body size perception

	Perceived thinness		Perceived fatness	
**a) Girls (n = 724)**				
Variable	OR	95% CI^a^	OR	95% CI^a^
Competitive goals in sports, yes vs no	0.88	0.38–2.06	0.86	0.53–1.39
BMI	0.56	0.45–0.70***	1.36	1.25–1.48***
Appearance/weight motives, per unit increase^b^	0.92	0.74–1.16	2.22	1.88–2.64***
Perceived physical fitness, good vs poor	0.57	0.28–1.17	0.30	0.19–0.47***
Health/fitness motives, per unit increase^b^	1.04	0.80–1.35	0.76	0.63–0.91**
**b) Boys (n = 687)**				
Variable	OR	95% CI^a^	OR	95% CI^a^
Competitive goals in sports, yes vs no	1.16	0.74–1.80	0.58	0.29–1.14
BMI	0.69	0.62–0.78***	1.46	1.31–1.61***
Appearance/weight motives, per unit increase^b^	0.90	0.75–1.07	1.58	1.26–1.99***
Perceived physical fitness, good vs poor	0.60	0.34–1.07	0.20	0.11–0.36***
Health/fitness motives, per unit increase^b^	1.05	0.88–1.25	0.82	0.64–1.04

“About the right size” was the reference category. The models were controlled for competitive goals in sports, BMI, perceived physical fitness, self-rated health, appearance/weight motives, enjoyment motives, health/fitness motives, social motives, and moderate to vigorous physical activity

*BMI* body mass index, *CI* confidence interval, *OR* odds ratio

^a^Significance of the OR: **p < 0.01 and ***p < 0.001

^b^Range between 0 and 6, a higher score indicating greater emphasis

## Discussion

In this study, competitive goal-oriented adolescents more frequently perceived their body as about the right size, perceived their physical fitness as higher, and rated their overall health as better than that of adolescents in the reference group. In multivariable analyses, competitive goals in sports were not associated with BSP. After adjustments, those with appearance/weight motives to exercise and poor perceived physical fitness had higher odds of having a perception of a too fat body. Additionally, BMI was positively associated with perceived fatness and negatively with perceived thinness.

To our knowledge, this is the first study concerning associations between adolescents’ competitive goals in sports and BSP. One recent study observed that adolescents in competitive sports had greater body appreciation and lower body dissatisfaction than non-athletes [18]. The authors found no difference between competitive athletes and leisure exercisers, and BSP did not differ between young competitive athletes, leisure athletes, and non-athletes [18]. Due to different measures, these previous findings cannot be directly compared with our results, but they are in line with the suggestion that adolescents’ BSP is mainly determined by other factors than having competitive goals in sports.

In accordance with a previous meta-analysis [8], our findings suggest that it is not having competitive goals, but rather the accompanying leanness and/or fitness that mediate the lower body image disturbance in competitive goal-oriented athletes than others. In addition, our results support the previous findings among adolescents on the association between higher BMI and body dissatisfaction [4, 31]. Gender differences have also been reported: the association between BMI and body dissatisfaction has been suggested to be linear in girls and U-shaped in boys [32]. We found that BMI was inversely associated with perception of a too thin body among both genders. Our finding is in line with a recent study which found that Finnish adolescents with thinness were more likely to wish for a bigger body than their normal-weight peers [33].

Perceived good physical fitness and health were more frequent among adolescents who perceived their body to be about the right size, and nearly all adolescents with competitive goals in sports self-reported good health and fitness. These findings are consistent with previous studies that have reported a relationship between poor perceived health and body dissatisfaction in adolescence [34]. Body and weight dissatisfaction does not seem to motivate healthy weight-management behaviours, but rather motivates unhealthy ones, such as disordered eating and lower levels of exercise and physical activity [35–37]. Our finding supports the current research among adults suggesting that perceived physical fitness is related to body image, and the association between physical activity and body image would be mediated via better perceived physical fitness [38].

Appearance and weight-related reasons to exercise have previously been associated with higher body dissatisfaction in adolescents and adults [39, 40]. In this study, adolescents with competitive goals in sports generally reported more importance placed on motives to exercise on nearly all measures, excluding weight control motives. Appearance and weight management as exercise motives were also positively associated with perceived fatness. This association remained in logistic regression analyses and was therefore found to exist independent of BMI as well. Additionally, girls who exercised to improve their health and physical fitness had lower odds of perceived fatness. One previous study among adolescents found no association between body dissatisfaction and positive reasons to exercise, such as improve health and body fitness, but girls and boys were analysed together [39].

Adolescents who had competitive goals in sports had higher MVPA than youths in the reference group, which may have confounded the association between having competitive goals in sports and BSP. After adjustments, physical activity was no longer associated with BSP in this study. It appears that among competitive goal-oriented adolescents, the actual weight status, perceived physical fitness, and appearance and weight-related motives to exercise also determine BSP more strongly than the amount of physical activity. A previous study among Finnish adolescents [36] also found that physical activity was not related to weight dissatisfaction among girls, but boys who were more physically inactive were more dissatisfied with their weight.

Fourteen percent of the girls were overweight in this study but half of the girls felt they were too fat. The ratio of perceived fatness and being overweight was higher among girls with competitive goals in sports than it was in the reference girls. This might suggest that although competitive goal-oriented girls generally view their weight more positively, they also experience more pressure to reach a certain leanness/fitness level or they may have a stricter body ideal. These findings are in line with the previous studies [13, 15], where body dissatisfaction was positively associated with competition level among collegiate female athletes. A similar effect was not found among boys in our study.

The strength of this study is that it is the first to evaluate the associations between adolescents’ competitive goals in sports and BSP among a representative sample of Finnish sports club participants and non-participants. The study contributes to our understanding of BSP among mid-adolescent athletes, whereas most previous studies on athletes’ body image have been conducted among older adolescents or adults. There are also some limitations in the present study. First, our study design was cross-sectional and does not allow differentiation between causes and consequences.

Second, our analysis was mainly based on self-report questionnaire data, where a large proportion of invited adolescents (60% of the sports club sample and 20% of the school-based sample) did not participate in the study. We have no details on non-respondents, but one previous Finnish study on adolescents’ weight perception [36] has suggested that adolescents who did not participate in the study were more often boys from families with low socioeconomic status, and therefore, boys’ body size dissatisfaction might be underreported.

Third, single-item measures were used both for competitive goals and BSP that might have simplified those complex constructs. Due to the diversity of goal orientations and BSPs, we recommend using validated questionnaires in future studies. Fourth, sports types, such as leanness-focused and non-leanness-focused sports, were not differentiated in this study. In future, the possible associations between various types of physical activity and adolescents’ BSP should be examined.

## Conclusions

BMI, importance placed on appearance/weight motives to exercise, and poor perceived physical fitness were more strongly associated with adolescents’ BSP than having competitive goals in sports. Perceived fatness in relation to being overweight was common among girls with competitive goals in sports that should be considered in youth organized sports. The role of competitive sports participation for body image development needs to be examined in qualitative and prospective studies using validated measures, especially in girls.

## Supplementary Information

**Additional file 1: Supplementary Table 1.** Motives to exercise by competitive goals in sports. **Supplementary Table 2.** Motives to exercise by body size perception.

## Data Availability

The datasets used and/or analysed during the current study are available from the corresponding author on reasonable request.
