# Supplementary material for: Factors associated with body size perception among adolescent goal-oriented sports participants and non-participants: a cross-sectional Finnish study
Source: BMC Public Health. 2022 Nov 25;22:2188. doi: 10.1186/s12889-022-14511-z (PMC9700987; doi:10.1186/s12889-022-14511-z)
Supplement: Supplementary file 1 — Additional file 1: Supplementary Table 1. Motives to exercise by competitive goals in sports. Supplementary Table 2. Motives to exercise by body size perception. [file 12889_2022_14511_MOESM1_ESM.docx]

**Supplementary material**

Supplementary Table 1. Motives to exercise by competitive goals in sports.

|  | Girls (n = 724) | | | | Boys (n = 687) | | | |
| --- | --- | --- | --- | --- | --- | --- | --- | --- |
|  | CGS (n = 199) | | RG (n = 525) | | CGS (n = 276) | | RG (n = 411) | |
| Appearance/weight motives |  |  |  |  |  |  |  |  |
| To look good | 185 | (93.0) | 479 | (91.2) | 258 | (93.5) | 335 | (81.5)*** |
| To control weight | 159 | (79.9) | 459 | (87.4)* | 185 | (67.0) | 304 | (74.0) |
| To lose weight | 84 | (42.2) | 353 | (67.2)*** | 56 | (20.3) | 176 | (42.8)*** |
| Health/fitness motives |  |  |  |  |  |  |  |  |
| To improve health | 197 | (99.0) | 506 | (96.4) | 269 | (97.5) | 385 | (93.7)* |
| To improve physical fitness | 199 | (100.0) | 507 | (96.6)* | 268 | (97.1) | 381 | (92.7)* |
| To gain muscle | 165 | (82.9) | 417 | (79.4) | 254 | (92.0) | 357 | (86.9)* |
| Social motives |  |  |  |  |  |  |  |  |
| To win | 181 | (91.0) | 231 | (44.0)*** | 269 | (97.5) | 255 | (62.0)*** |
| To be good in sports | 198 | (99.5) | 430 | (81.9)*** | 276 | (100.0) | 347 | (84.4)*** |
| To make new friends | 195 | (98.0) | 431 | (82.1)*** | 256 | (92.8) | 331 | (80.5)*** |
| To meet my friends | 197 | (99.0) | 464 | (88.4)*** | 264 | (95.7) | 365 | (88.8)** |
| To please my parents | 69 | (34.7) | 147 | (28.0) | 99 | (35.9) | 176 | (42.8) |
| To be “cool” | 67 | (33.7) | 131 | (25.0)* | 127 | (46.0) | 186 | (45.3) |
| Enjoyment motives |  |  |  |  |  |  |  |  |
| To have fun | 199 | (100.0) | 498 | (94.9)** | 274 | (99.3) | 389 | (94.6)** |
| To appreciate the sensations during exercise | 198 | (99.5) | 466 | (88.8)*** | 269 | (97.5) | 356 | (86.6)*** |
| Exercise is exhilarating | 196 | (98.5) | 423 | (80.6)*** | 260 | (94.2) | 346 | (84.2)*** |

CGS = competitive goals in sports; RG = reference group. Values are presented as number of participants (%). *P* values are presented for differences between CGS and reference groups: *p < 0.05, **p < 0.01, ***p < 0.001.

Supplementary Table 2. Motives to exercise by body size perception.

|  | Girls (n = 724) | | | | | | Boys (n = 687) | | | | | |
| --- | --- | --- | --- | --- | --- | --- | --- | --- | --- | --- | --- | --- |
| Body size perception | Too thin^a^  (n = 49) | | About the right size  (n = 379) | | Too fat^b^  (n = 296) | | Too thin^a^  (n = 130) | | About the right size  (n = 454) | | Too fat^b^  (n = 103) | |
| Appearance/weight motives |  |  |  |  |  |  |  |  |  |  |  |  |
| To look good | 40 | (81.6) | 342 | (90.2) | 282 | (95.4)* | 111 | (85.4) | 396 | (87.2) | 86 | (83.5) |
| To control weight | 31 | (63.3)* | 303 | (79.9) | 284 | (95.9)*** | 68 | (52.3)*** | 327 | (72.0) | 94 | (91.3)*** |
| To lose weight | 10 | (20.4)** | 155 | (40.9) | 272 | (91.9)*** | 21 | (16.2)** | 128 | (28.2) | 83 | (80.6)*** |
| Health/fitness motives |  |  |  |  |  |  |  |  |  |  |  |  |
| To improve health | 48 | (98.0) | 369 | (97.4) | 286 | (96.6) | 119 | (91.5)* | 436 | (96.0) | 99 | (96.1) |
| To improve physical fitness | 45 | (91.8)* | 372 | (98.2) | 289 | (97.6) | 121 | (93.1) | 432 | (95.2) | 96 | (93.2) |
| To gain muscle | 42 | (85.7) | 295 | (77.8) | 245 | (82.8) | 118 | (90.8) | 402 | (88.5) | 91 | (88.3) |
| Social motives |  |  |  |  |  |  |  |  |  |  |  |  |
| To win | 31 | (63.3) | 227 | (59.9) | 154 | (52.0)* | 99 | (76.2) | 357 | (78.6) | 68 | (66.0)** |
| To be good in sports | 45 | (91.8) | 335 | (88.4) | 248 | (83.8) | 117 | (90.0) | 420 | (92.5) | 86 | (83.5)** |
| To make new friends | 42 | (85.7) | 338 | (89.2) | 246 | (83.1)* | 103 | (79.2)** | 403 | (88.8) | 81 | (78.6)** |
| To meet my friends | 46 | (93.9) | 352 | (92.9) | 263 | (88.9) | 118 | (90.8) | 422 | (93.0) | 89 | (86.4)* |
| To please my parents | 15 | (30.6) | 99 | (26.1) | 102 | (34.5)* | 49 | (37.7) | 183 | (40.3) | 43 | (41.7) |
| To be “cool” | 21 | (42.9)** | 89 | (23.5) | 88 | (29.7) | 58 | (44.6) | 209 | (46.0) | 46 | (44.7) |
| Enjoyment motives |  |  |  |  |  |  |  |  |  |  |  |  |
| To have fun | 47 | (95.9) | 370 | (97.6) | 280 | (94.6)* | 123 | (94.6) | 444 | (97.8) | 96 | (93.2)* |
| To appreciate the sensations during exercise | 44 | (89.8) | 357 | (94.2) | 263 | (88.9)* | 118 | (90.8) | 421 | (92.7) | 86 | (83.5)** |
| Exercise is exhilarating | 42 | (85.7) | 333 | (87.9) | 244 | (82.4)* | 115 | (88.5) | 409 | (90.1) | 82 | (79.6)** |

Values are presented as number of participants (%). ^a^*P* values for difference between the groups “too thin” and “about the right size”: *p < 0.05, **p < 0.01, ***p < 0.001. ^b^*P* values for difference between the groups “too fat” and “about the right size”: *p < 0.05, **p < 0.01, ***p < 0.001.
